# PID++: A Computationally Lightweight Humanoid Motion Control Algorithm

**DOI:** 10.3390/s21020456

**Published:** 2021-01-11

**Authors:** Thomas F. Arciuolo, Miad Faezipour

**Affiliations:** 1 Department of Computer Science & Engineering, University of Bridgeport, Bridgeport, CT 06604, USA; tarciuol@my.bridgeport.edu; 2 Department of Biomedical Engineering, University of Bridgeport, Bridgeport, CT 06604, USA

**Keywords:** adaptive motion control, PID++ algorithm, humanoid, computationally lightweight

## Abstract

Currently robotic motion control algorithms are tedious at best to implement, are lacking in automatic situational adaptability, and tend to be static in nature. Humanoid (human-like) control is little more than a dream, for all, but the fastest computers. The main idea of the work presented in this paper is to define a radically new, simple, and computationally lightweight approach to humanoid motion control. A new Proportional-Integral-Derivative (PID) controller algorithm called PID++ is proposed in this work that uses minor adjustments with basic arithmetic, based on the real-time encoder position input, to achieve a stable, precise, controlled, dynamic, adaptive control system, for linear motion control, in any direction regardless of load. With no PID coefficients initially specified, the proposed PID++ algorithm dynamically adjusts and updates the PID coefficients Kp, Ki and Kd periodically. No database of values is required to be stored as only the current and previous values of the sensed position with an accurate time base are used in the computations and overwritten in each read interval, eliminating the need of deploying much memory for storing and using vectors or matrices. Complete in its implementation, and truly dynamic and adaptive by design, engineers will be able to use this algorithm in commercial, industrial, biomedical, and space applications alike. With characteristics that are unmistakably human, motion control can be feasibly implemented on even the smallest microcontrollers (MCU) using a single command and without the need of reprogramming or reconfiguration.

## 1. Introduction

### 1.1. Motivation and Background

Today, motion control can be found in many aspects of every-day life, including items purchased at stores, as well as systems used in industry, medicine and space. Computer printers, fax machines, robot arms and robotic surgery are a few examples of such devices and applications. Some of the applications, such as printers and fax machines, travel gracefully from place to place, but their motion is static. If the specifications of the system such as its weight or size change, the device’s operation will probably come to a grinding halt.

**Figure 1:**
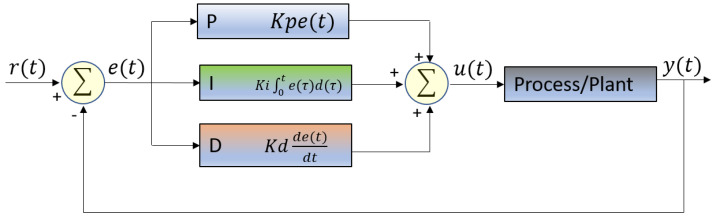
Basic PID block diagram.

Humans are not burdened by a requirement to move only one particular object all of the time. A printer’s carriage motor, for example, is. Its control program will never move anything else. Humans can move, pickup, and put down lightweight objects and heavy objects alike. Doing so is automatic and effortless. Today’s industrial robots can do this accurately as well, unfortunately only with extremely computationally costly algorithms running on high-speed computers. Motion control on a low-end computing device, with an algorithm such as the Proportional-Integral-Derivative (PID), will allow the controlled motion of only one particular object at a time. To move another object, perhaps heavier or lighter, with the same algorithm, would more than likely require at least the reprogramming of the PID coefficients (Kp, Ki, Kd), and the like. Figure 1 shows the basic PID loop [1], where static PID coefficients are determined to compute the output control function u(t) based on Equation (1) according to the error e(t).

(1) $$u\left(t\right)=Kp\times e\left(t\right)+Ki\times \int_{0}^{t}e(\tau )d(\tau )+Kd\frac{de(t)}{dt}$$

Dynamic, on the fly adaptability are hallmarks of human motion control. With the algorithm presented in this paper, called the “PID++”, not only is this dynamic adaptability of human-like (humanoid) control present, but also positional control with accuracy down to 0.0001”, as well as speed and acceleration control, typically found in expensive servo systems. The PID++ algorithm assumes a fully-specifiable, trapezoidal and symmetrical, acceleration and deceleration travel profile.

Linear motion control is the moving of an object from Point A to Point B, also known as “a run”. There are 4 major classifications of linear motion control [2]:

1. Type 1: Moving an object perpendicular to gravity with friction, whereupon the de-application of power, causes the motion to stop. A printer carriage is an example of this Type 1 motion.
2. Type 2: Moving an object perpendicular to gravity without friction (such as on a friction-less table or in outer space). In this scenario, the de-application of power does not cause a slow-down of the object, but slow-down and stopping is affected by applying reverse power to the motor drive. A jet airplane landing on a runway, where reverse thrust is applied to the jets to affect a slow-down, and stop of the airplane, is an example of this Type 2 motion.
3. Type 3: Moving an object parallel to gravity upward and against the force of gravity, i.e., lifting an object. A weight-lifting machine at the gym is an example of an apparatus that exhibits this Type 3 motion, where the user lifts a weight.
4. Type 4: Moving an object parallel to gravity downward and with the force of gravity, i.e., the controlled decent of an object. A weight-lifting machine at the gym is an example of an apparatus that exhibits this Type 4 motion, where the user, in a controlled fashion, brings the weight to the bottom, on the platform.

There are many examples of each of these 4 types of linear motion control, and other examples of compound types of linear motion control.

### 1.2. Humanoid Motion Control—Problem Identification

We state the research problem definition of humaniod motion control by a single illustrative example. Assume a blindfolded person sits in front of a weight lifting machine at the gym and is asked to lift an unknown weight from the bottom of the platform to a specific height at a particular speed and with a particular trapezoidal, acceleration and deceleration profile. The person must at all times comply with this request, and has no foreknowledge of the amount of weight to be lifted (Type 3). After this run is completed, the user must do the same, with this same weight, or an unknown different amount, and bring this weight back down to the bottom of the platform (Type 4), while always maintaining compliance to the motion specifications. The person will do this repeatedly with many different amounts of weight, one after another. How would the person approach this problem? What would the human/machine operation look like?

The way a human would approach this problem, i.e, the human-like operation of the motion is referred to as humanoid motion control in this paper. It is clear that a human would apply more force when sensing heavier weights to be lifted, and may possibly exhibit slight vibrations at the end of the run (up-lifting motion), that generally corresponds to searching for the target at the end of the run.

This is a problem addressed by the PID++ algorithm, and more generally, addressing all four (4) motion control types with a single algorithm, while not requiring any sort of adjustment or reconfiguration. Additionally, the algorithm must be computationally lightweight, and as such, be able to execute well on even a small microcontroller (MCU) such as the Arduino Uno.

### 1.3. Key Contributions

This article proposes to overcome the existing hurdles of motion control systems tailored for specific applications to exhibit a rather generalized computationally lightweight humanoid motion control behavior. The basic PID loop is generally used for the implementation of motion control but suffers from having only static coefficients. The main idea of the work presented in this paper is to define a radically new, simple, and computationally lightweight approach to humanoid motion control, which has the basic PID at its heart. A new PID controller algorithm called PID++ is proposed in this work that uses minor adjustments with basic arithmetic, based on the real-time encoder position input, to achieve a stable, precise, controlled, dynamic, adaptive control system, for linear motion control, in any direction regardless of load.

The proposed PID++ algorithm is able to perform any of the 4 types of motion control, independent of weight, all without any reprogramming, adjustments or foreknowledge/transfer function, of any kind, just like humans do all the time, but, with the speed, precision, and accuracy of a computer controlled machine. The algorithm is even able to cope with changes in load during the middle of a run.

The PID++ assumes linearity of control and that there will be no delays in the control feedback, encompassing the majority of motion control applications. The machine executing the PID++ algorithm will never be asked to run with loads beyond its mechanical abilities.

With basic arithmetic, minor incremental adjustments based on real-time input, has been made to achieve a stable, controlled, dynamic, adaptive control system, for linear motion control, in any direction and with any load. With no PID coefficients initially specified, the proposed PID++ algorithm dynamically adjusts and updates the PID coefficients Kp, Ki and Kd periodically. In addition, no database of values is required to be stored as only the current and previous values of the sensed position with an accurate time base are used in the computations and overwritten in each read interval, eliminating the need of deploying much memory for storing and using vectors or matrices. The simplicity of the basic arithmetic computations, done periodically as the run progresses, makes the algorithm computationally lightweight. The PID++ algorithm allows adaptive motion control to be feasibly implemented on even the smallest microcontrollers (MCU) using a single command and without the need of reprogramming or reconfiguration. In this article, the implementation of the PID++ algorithm is done on a simple Arduino Uno.

The PID++ is designed to be a complete motion control algorithm handling every detail of the motion control run from start to finish, and does so with the human-like characteristics of adaptability and coping with adverse conditions, all in real time. The implementation, results and comparisons support these contribution claims.

### 1.4. Paper Organization

The remainder of this paper is organized as follows. Section 2 glances at prior related work in the literature regarding motion control. The development of the proposed solution is described in Section 3. The proposed PID++ algorithm is explained in detail in Section 4. The experimental results for various scenarios and runs are presented in Section 5. Comparison discussion, computational complexity analysis, limitations of the algorithm and applications of the proposed work are also presented later in Section 5. Finally, concluding remarks and future directions appear in Section 6.

## 2. Related Work

For as long as humans have been on planet Earth, people have naturally and effortlessly been able to control their neural motor activities to be able to interact and shape the world around them. Today, the question is: can an algorithm be devised to endow a machine with the same degree of motor control as that of a single muscle of the human body controlled by the human brain?

To date, the work in this field [3,4,5,6,7,8,9,10,11,12,13] is primarily divided into four categories. All are computationally intensive, and none offer a unified approach to complete motion control. The four categories are:

(a) Single Neuron [3,4,5];
(b) Fuzzy Logic [6,7,8,9,10,11];
(c) Self-Tuning [12,13];
(d) Model Reference (based on the Laplace Transform) [13].

What is most notable about all of these references is that each is geared to a particular application, and not for generalized motion control. Moreover, they all run on either high-speed CPUs such as a Digital Signal Processor (DSP) or specialized hardware.

The work in [3] uses a Single Neuron to make PID adjustments for the motion of a mechanical arm. The work, however, focuses on specifically the noted application. The authors in [4] proposed to improve the Single Neuron PID approach using Fuzzy Logic gain control, focusing on nonlinear and time delay control. While there is no doubt a need for nonlinear and/or time delayed control, it is noteworthy to mention that 90% of motion control is with linear systems that do not have any delays in the control feedback [14]. The authors in [5] use a Single Neuron approach to tune the PID, only to control the lateral motion of an automobile driving down a road. The authors of these Single Neuron based designs achieve plausible results for the intended motion control applications. However, the computational complexity of such designs are high, making it impractical to be implemented on lightweight controllers, especially if retraining is required.

The idea presented in [6] is a simulation study of the comparison of the classical PID with a Fuzzy Logic tuned PID related to hydraulics. The work presented credible results, but is tailored for the very specific application of hydraulics. The authors in [7] use a high-speed Application Specific Integrated Circuit (ASIC) to implement the Fuzzy Logic controlled tuning of the PID, strictly for aircraft roll control. The computational complexity and costume-logic requirements of this design for aircraft roll control calls for implementation in specialized hardware devices such as ASICs, which are not reprogrammable or reconfigurable. In [8], the authors use a Digital Signal Processor (DSP) to execute a Fuzzy Logic based self-tuned PID to overcome servo deficiencies such as nonlinearity, and the servo lag phenomena. DSP processors allow for complex and nonlinear math functions required for controlling servo systems, but rather fall in the category of computationally complex (heavy) devices. The authors in [9] use Fuzzy Logic to tune the PID for improved performance in the robot soccer game. The work in [10] is another Fuzzy Logic attempt to tune a PID for improved performance in the game of robot soccer. These work focus on the specific robot soccer game application and obtain reasonable results, but cannot be generalized for other motion control applications. The work in [11] is a Fuzzy Logic approach to tuning a PID for purposes of compensating for increased friction on a solar tracker. The work relies on specifying the fuzzy rules for motion control particularly applied to a solar tracker, and thus would require defining new rules for other types of motion control.

The authors in [12] use traditional Self-Tuning of the PID controller on a high-end DSP to improve robot motion control. The idea of [13] is another attempt at PID adjustment for aircraft roll control by using two different approaches to tune the PID to improve performance: (1) traditional Self-Tuning, and (2) using a predefined model (Laplace System Transfer Function) as a means to adjust the PID. These approaches deal with the transfer function of the process/system as well as the PID functions in the Laplace mathematical domain. For such methods, the specific application along with the Laplace transform of the control process should be known, or computed prior to adjusting the PID functionality. These techniques cannot perform with an unknown control process Laplace transform (referring to the process/plant box in Figure 1), and thus are not suitable for generalized motion control.

To achieve robustness, other related work such as [15,16] discuss the use of evolutionary algorithms such as particle swarm optimization (PSO), artificial bee colony (ABC) and cuckoo search algorithms to optimize PID tuning under internal and external disturbances. These work present promising results for PID adjustment, however focus on specific applications such as automatic voltage regulator (AVR) with time delay.

As can be seen, generalized motion control that can be embedded in low-power and computationally lightweight hardware has not been thoroughly investigated in the literature. This paper, however, aims to tackle this problem in more detail. The proposed algorithmic approach in this paper is the simplest and most dynamically adaptive technique for PID self-tuning, providing a complete motion control solution, unlike the prior related work [3,4,5,6,7,8,9,10,11,12,13] and other algorithms [17,18,19,20,21,22,23], for generalized humanoid motion control.

## 3. Development of the Proposed Solution

In this paper, an algorithm is devised to approach the problem of obtaining human-like motion control operation using a low-end microcontroller, an Arduino Uno, an Arduino Motor Control Shield, Encoder Pulse Counter Shield, Gear-Motor with Integrated Encoder, Pulley Wheel, Run Start Button, and a Laboratory-grade Test Fixture with Assorted Precision Weights. The algorithm is initially developed with the aid of MATLAB simulation and MATLAB is also used for graphical output to report the algorithm responses and results of operation based on data from actual runs.

**Figure 2:**
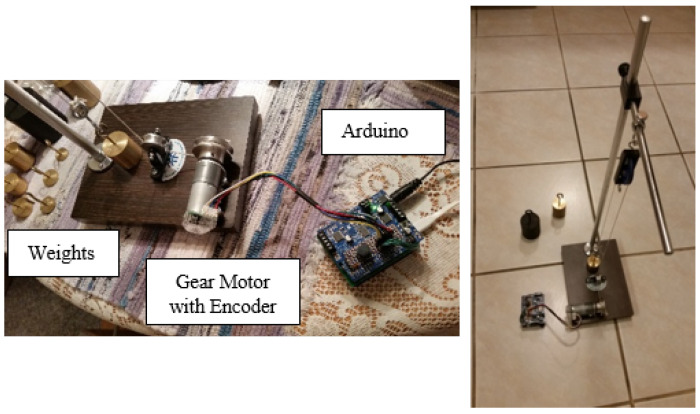
PID++ development and test apparatus.

Additionally, real-time data from actual runs are reported in the Arduino IDE for detailed analysis. The actual motor operation in the Arduino IDE is coded in C programming language. The development platform and test apparatus are shown in Figure 2.

## 4. PID++ Algorithm Design

**Figure 3:**
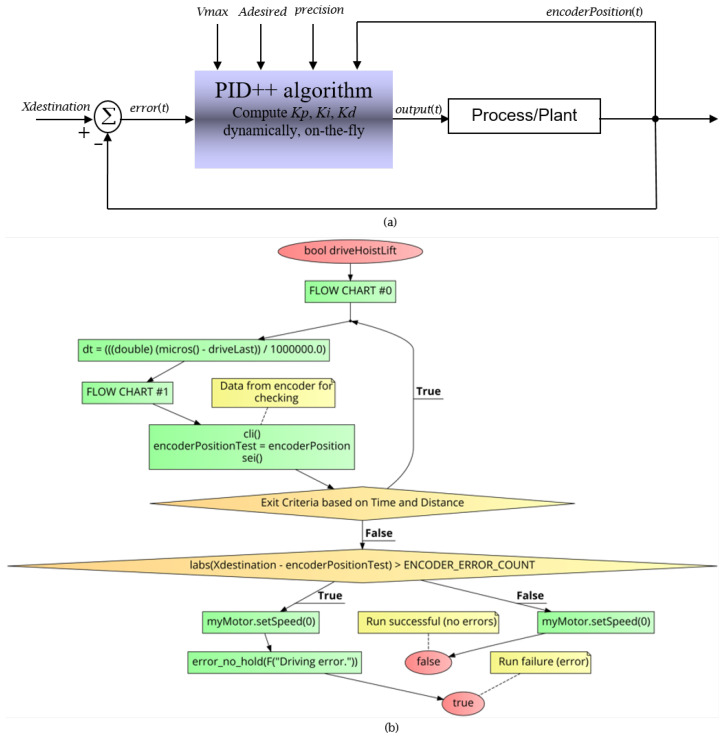
(**a**) PID++ control block diagram. (**b**) PID++ Flow Chart—Main Level.

The PID++ algorithm is designed to provide a complete holistic solution to motion control. With a single command, a full run, from beginning to end, is executed. Figure 3a shows the overall control block diagram and Figure 3b shows the MAIN LEVEL FLOW CHART, command line parameters and operation of the PID++ algorithm.

Like the basic PID, the following Equations (2)–(6) are used. However, the output of the PID++ algorithm expressed in Equation (2) at time *t*, is computed based on dynamic Kp, Ki and Kd coefficients, not initially specified.

PID Equations:

(2) output=Kp×error+Ki×integral+Kd×derivative

where,

(3) error=Xdesination−Xcurrent

where,

(4) Xcurrent=encoderPosition

(5) integral=integral+error×dt

(6) derivative=(error−previous_error)/dt

The PID++ routine is called with a destination value (Xdesination in encoder counts (or cnts), e.g., 125,000), a plateau travel speed (Vmax in counts/ms), an acceleration (Adesired in counts/ms${}^{2}$), a minimum time granularity (dtmin in seconds, e.g., 0.005) specifying an algorithm recalculation time interval, and a physical quantization granularity tolerance called precision (unitless, e.g., 0.01), as the user specified inputs. The basic PID computations are recalculated every dtmin along with all three (3) PID coefficients Kp, Ki and Kd. Encoder feedback is what drives this algorithm to control every aspect of its operation.

The MAIN LEVEL FLOW CHART (Figure 3b) presents the command line parameters of the PID++ algorithm in a C-like pseudo-code structure for lifting up or bringing down weights in a humanoid controlled motion fashion. The bool driveHoistLift function takes the PID++ Xdesination, Vmax, Adesired, dtmin and precision user input parameters. Other inputs such as the encoderPosition are sensed every dt time intervals. In real time applications implemented in hardware, precise dt can be computed by keeping track of the current time with accuracies in the range of microseconds. Additional parameters such as the hold time for lift and maximum allowable drive interval are defined here using the ENCODER_ERROR_COUNT variable.

After variable initialization and first-pass execution through FLOW CHART #0, the program enters the main loop until the destination is reached but for no longer than a maximum allowable drive interval. In this loop of the algorithm, if the minimum time granularity has been reached, then, FLOW CHART #1 is executed, otherwise and always, the current position is checked (sensed) to determine if the destination has been reached within a target number of counts. The output is the quantified pulse width modulation (PWM) value driving the motor. This value should be within the motor’s strength and mechanical capability (PWM_LIMIT).

**Figure 4:**
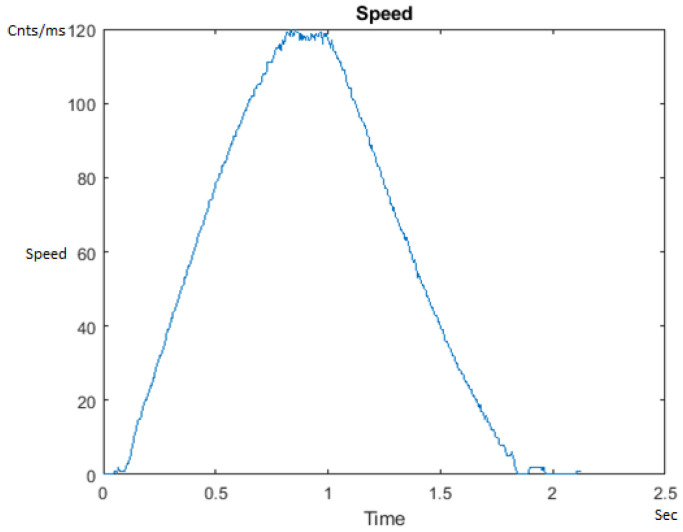
Speed graph of a typical run with PID++ motion control.

To develop the PID++ algorithm, we conceptualize how a typical run from one point to another (e.g., from the bottom of the platform to the top) should look like. Consider Figure 4 as a typical graph of the Speed of a run for Vmax = 120 counts/ms. The slope of the curve in different areas determines the acceleration.

The first thing to note is that the graph in Figure 4 is horizontally symmetrical about the center line of the graph to form the two (2) halves of the run. Now, there are four (4) main areas of the Speed graph (going from left to right in blue) to form a symmetrical trapezoid. The first is the linear acceleration zone up to the velocity of 120 counts/ms. Next comes the flat (constant velocity) plateau phase at 120 count/ms. From here we enter the end-of-run deceleration zone which gets us very close to the Xdestination. Finally, there is the search phase which brings the run to Xdestination within a tolerance as a function of the lifted weight. The heavier the weight for a given motor size, the looser the tolerance that is required for stable operation.

With this background, there are three (3) major aspects that comprise the PID++ algorithm:

1. The “Phase” structure;
2. The 3-Dimensional Polynomial;
3. The “holdCount” computation for the End of Run Deceleration Zone.

The structure of the PID++ algorithm is explained hereafter in more detail through describing the these three main aspects along with the related illustrated flow charts.

### 4.1. The Phase Structure

In terms of the detailed operation, the PID++ algorithm has nine specific areas of operation called phases. These phases correlate to the “phase” of the run that the algorithm is in the process of executing:

- Phase 0: Initialization and first pass at time = 0. This is where the run preparations are made and initial setup is carried out.
- Phase 1: First half of run, velocity is too low. This points to the initial acceleration zone trying to reach the plateau phase.
- Phase 2: First half of run, velocity is too high. In this case, the system is not yet ready to begin the plateau phase but somehow managed to overshoot the Vmax.
- Phase 3: First or second half of run, velocity has plateaued, and running at the specified velocity (Vmax).
- Phase 4: Second half of run, before the end of run deceleration zone, plateaued, but velocity has become too low over time. This refers to the plateau phase but traveling at a speed below tolerance (Vmax−precision).
- Phase 5: Second half of run, before the end of run deceleration zone, plateaued, but velocity has become too high over time. This is in the plateau phase but traveling at a speed above tolerance (Vmax+precision).
- Phase 6: End of run deceleration zone. This is where the system is ready to begin decelerating to the Xdestination.
- Phase 7: Projected velocity at the destination is outside of “precision”. This refers to when the system is decelerating to the target but the projected speed at the target is not within “precision”.
- Phase 8: Projected velocity at the destination is within “precision”. This refers to when the system is decelerating to the target and the projected speed at the target is within “precision”.

### 4.2. The 3-Dimensional Polynomial

The “div” 3-dimensional polynomial is the f(Vmax) polynomial and the g(Adesired) conjoined by subtraction. This “div” value refers to the division of time for Kp, Ki and Kd adjustments. By properly dividing time for a specified Vmax and Adesired, a stable and accurate response is obtainable. This 3-dimensional polynomial is derived empirically and is scalable. This value is calculated at the beginning of the run in Phase 0, and is static until the run reaches the end of run deceleration zone, where it then becomes dynamic (due to an augmentation carried out in the third aspect of the PID++ algorithm).

Upon exiting the main loop (MAIN LEVEL FLOW CHART), if the destination has been reached, the PID++ routine returns success otherwise the routine returns failure.

**Figure 5:**
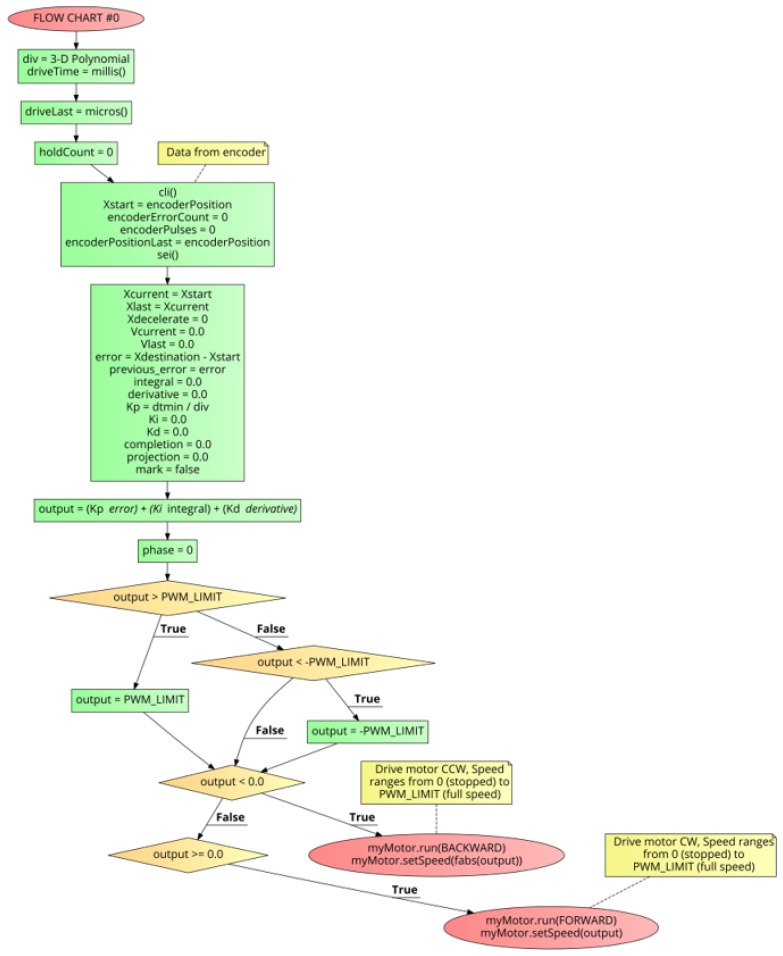
PID++ Flow Chart #0 - Initialization Stage.

**Figure 6:**
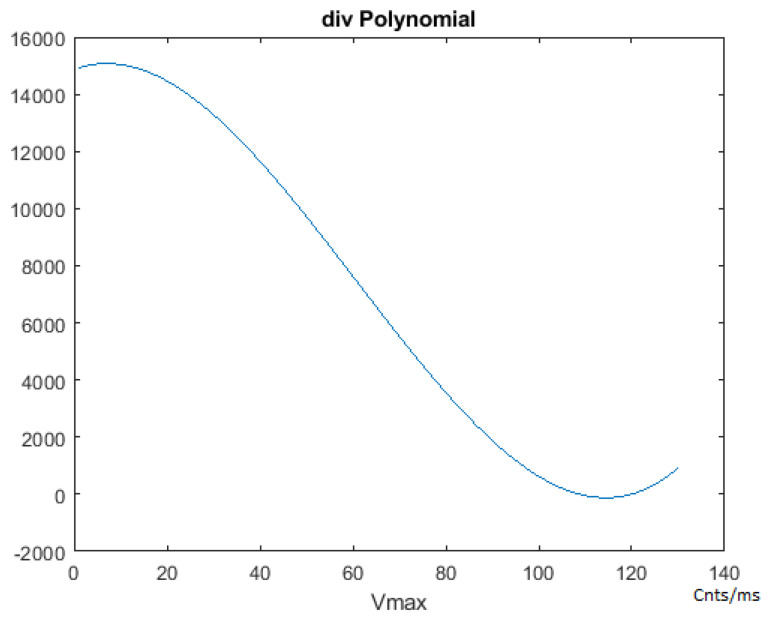
PID++ Vmax polynomial.

**Figure 7:**
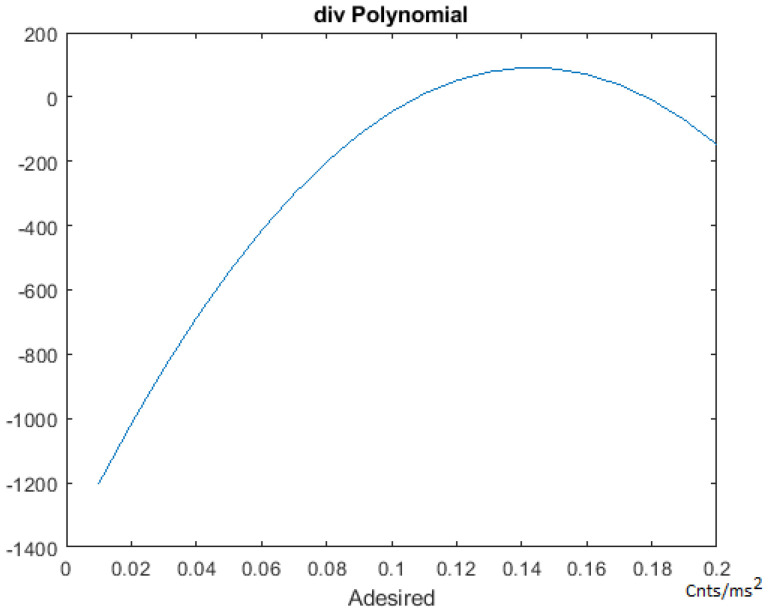
PID++ Adesired polynomial.

Figure 5 shows FLOW CHART #0. The first step of this section is the one-time calculation of the “div” parameter through the execution of a 3-dimensional polynomial (Equations (7)–(9)). This polynomial takes the Vmax and Adesired parameters as inputs. See Figure 6 and Figure 7.

(7) div=f(Vmax)−g(Adesired)

(8) $$\begin{array}{cc} \; & f(Vmax)=(14871.428374977424\;+(Vmax\times 58.963462093835631)\\ \; & \;\;\;\;\;\;\;\;\;\;\;+(Vmax^{2}\times \left(-4.4655757092373358\right))\\ \; & \;\;\;\;\;\;\;\;\;\;\;+(Vmax^{3}\times 0.024514638540135931)) \end{array}$$

(9) $$\begin{array}{cc} \; & g(Adesired)=(-1402.4961019577061\;\;\;\;\;+(Adesired\times 20724.637961900153)\\ \; & \;\;\;\;\;\;\;\;\;\;\;\;+(Adesired^{2}\times \left(-70942.626241111837\right))\\ \; & \;\;\;\;\;\;\;\;\;\;\;\;+(Adesired^{3}\times \left(-6557.7905889448139\right)) \end{array}$$

This “div” variable is used in the on-the-fly tuning process of the PID++, for updating the PID coefficients, performed in real-time throughout the run. These equations have been developed empirically with much data taken in actual runs. From all these data, the “div” parameter equations were derived using regression and curve fitting models.

The f(Vmax) portion of the “div” value relative to a precise time base “dt” allows for the proper update rate of the basic PID coefficients to affect a desired plateau speed. The slope of the trapezoidal acceleration and deceleration is affected by the g(Adesired) portion of the “div” variable by augmenting the way the f(Vmax) functions, by either enhancing or retarding its effect. By doing so, the slope of the trapezoid is affected on the way to the Vmax speed and from the Vmax speed back to stop motion.

The coefficients of the polynomials are constant values found after curve fitting where the variables of the polynomials are the Vmax and Adesired input arguments. The div computation is done just once at the beginning of the program and is independent of the motor process, transfer function or load weights. Therefore, this developed 3D polynomial of div is used consistently the same in way in any run, and only depends on the Vmax and Adesired input values. By making small adjustments as needed on the way to the destination on a periodic basis, minor modification in real-time is all that needs to be done. This can be done with simple arithmetic, thus with a low computational cost.

After this “div” calculation, the rest of FLOW CHART #0, is the first pass execution of the PID++ algorithm, at time = 0. This code does an initialization of running variables and PID coefficients at this point in time. From here, the first PID calculation is made and outputs (Equations (2)–(6)), held within fixed limits, are sent to the motor to begin the run.

**Figure 8:**
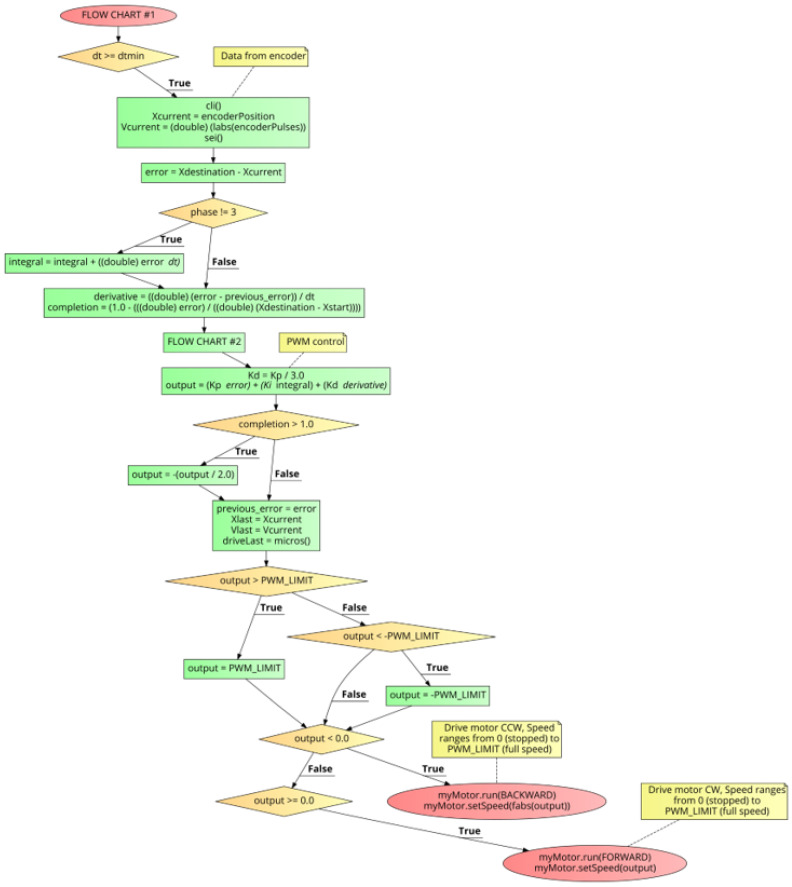
PID++ Flow Chart #1—output control level.

Figure 8 shows FLOW CHART #1 which shows the output control structure of the PID++ algorithm which is executed periodically in the Main Loop, every dtmin. At this programming level, a set of PID coefficients (Kp, Ki, Kd) have already been set. Using these current coefficient values, the running output (Equation (2)) is determined and used to control the motor, with adjustments made every dt depending on the phase of the run.

FLOW CHART #1 begins by confirming that at least a dtmin time period has elapsed since the last execution of this flow chart. If so, the current position and velocity is retrieved from the encoder. The “error” used with the proportional term of the PID is calculated (Equations (3) and (4)). If the algorithm is not currently in Phase 3, the integral term is computed (Equation (5)). However, if the algorithm is in Phase 3, no adjustment in the integral term is necessary. Now, the derivative (Equation (6)) and run “completion” fraction (Equation (10)) is calculated.

(10) $$\begin{array}{cc} \; & completion=1-\frac{error}{\left(Xdestination-Xstart\right)}\\ \; & \;\;\;\;\;=\frac{\left(Xcurrent-Xstart\right)}{\left(Xdestination-Xstart\right)} \end{array}$$

FLOW CHART #2 is then executed to determine any retuning of the PID coefficients. As a programmatic simplification, Kd=Kp/3.0 is used. With all variables now updated, the PID calculation is made and outputs (Equation (2)), held within fixed limits, are sent to the motor for the current iteration of the run, and repeated for the duration of the run. If the motor should overshoot the destination, the output is negated and attenuated as a means to drive the motor back to the precise destination target (Xdestination), within a fixed count tolerance.

**Figure 9:**
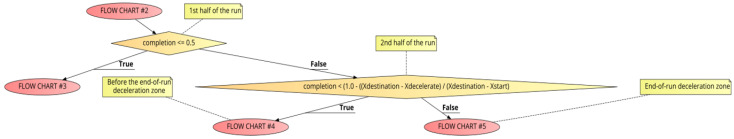
PID++ Flow Chart #2—completion control level.

Figure 9 shows FLOW CHART #2 which begins the low-level coefficient tuning code. The flow chart that gets executed next (FLOW CHART #3, #4, or #5), is determined in this section of the code based on the current value of the run “completion” fraction (Equation (10)).

**Figure 10:**
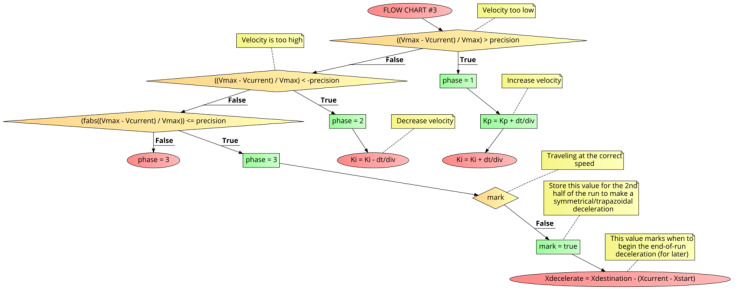
PID++ Flow Chart #3—first half of run (acceleration and plateau).

Figure 10 shows FLOW CHART #3 for the retuning of the PID coefficients during the first half of the run. The objective of the first half of the run is to accelerate the motor at the Adesired specification to the Vmax velocity and plateau there into the second half of the run, and make adjustments if necessary to maintain Vmax within “precision”.

Key to the computationally lightweight nature of the PID++ algorithm, is small adjustments done with just basic arithmetic.

Using the “div” value calculated only once at the beginning of the run, based on the 3-dimensional polynomial, which is a confluence of Vmax and Adesired into this single floating point number “div”, the Kp and Ki coefficients are given minor adjustment as needed.

In Figure 10, if the current velocity (Vcurrent) is under Vmax, as would be the case during the initial acceleration of the run, Kp and Ki are increased by “dt/div” (Kp=Kp+dt/div, Ki=Ki+dt/div). Once the Vmax speed is reached, the position is marked (Xdecelerate is calculated for the 2nd half of the run to form a symmetrical trapezoid acceleration/deceleration profile). Should there be an over-speed situation, only Ki is decreased by “dt/div” (Ki=Ki−dt/div) and Kp is left unchanged. During the plateau phase while running at Vmax to within “precision”, no adjustments to the Kp or Ki coefficients are made.

**Figure 11:**
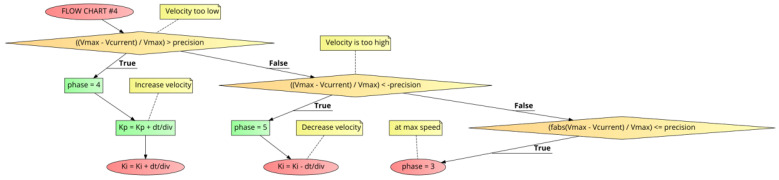
PID++ Flow Chart #4—second half of run (plateau before the end of run deceleration zone).

Figure 11 shows FLOW CHART #4 which continues the plateau through the 2nd half of the run, up to the end of run deceleration zone begun at Xdecelerate. If Vcurrent is under Vmax, Kp and Ki are increased by “dt/div” (Kp=Kp+dt/div, Ki=Ki+dt/div). Should there be an over-speed situation, only Ki is decreased by “dt/div” and Kp is left unchanged. During this plateau phase while running at Vmax to within “precision”, no adjustments to the Kp or Ki coefficients are made.

### 4.3. The “holdCount” for the End of Run Deceleration Zone

**Figure 12:**
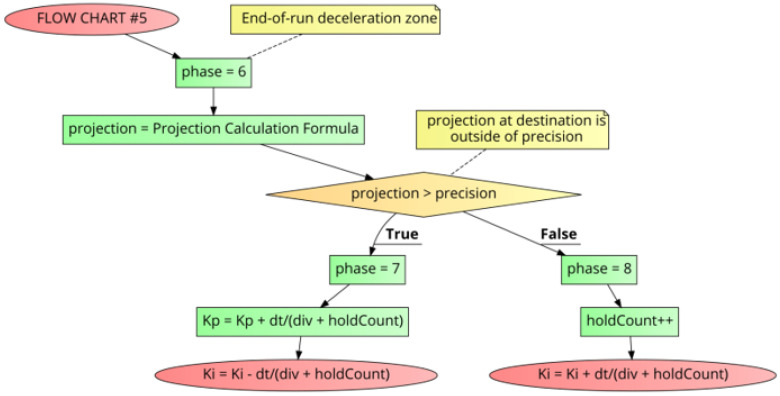
PID++ Flow Chart #5—second half of run (end of run deceleration zone).

During the run, Xdecelerate is the point where the motor has completed most of the run and must decelerate to a stop by the time it hits the Xdestination. Figure 12 shows FLOW CHART #5 which begins at the Xdecelerate point in the run. To perform proper deceleration, a “projection” calculation of the speed is made by looking at the deceleration rate (slope) relative to the remaining distance to travel to get to the Xdestination (Equation (11)).

(11) $$\begin{array}{cc} \; & projection=abs(\frac{\left(Vcurrent-Vlast\right)}{\left(Xcurrent-Xlast\right)}\times Xdestination\\ \; & \;\;\;+Vcurrent-\frac{\left(Vcurrent-Vlast\right)}{\left(Xcurrent-Xlast\right)}\times Xcurrent) \end{array}$$

If at this point in time, the “projection” is within the “precision” then a “holdCount” variable (which is only used in this section of the code) counts the number of times the “projection” is within the “precision”. This “holdCount” value, which can increase as the motor approaches the destination, is used to dynamically augment the statically calculated “div” value, derived from the 3-dimensional polynomial. In this case, Kp is untouched, and Ki is then increased by “dt/(div+holdCount)”. That is, Ki=Ki+dt/(div+holdCount).

If on the other hand, the “projection” is outside of the “precision”, then Kp is increased by “dt/(div+holdCount)”, and Ki is decreased by “dt/(div+holdCount)”. That is, Kp=Kp+dt/(div+holdCount), Ki=Ki−dt/(div+holdCount).

### 4.4. Overall PID++ Algorithm Behavior

The PID++ algorithm periodically senses the encoderPosition and computes the PID++ output based on periodic adjustments of Kp, Ki and Kd parameters to reach the destination in a humanoid controlled motion fashion. The current velocity Vcurrent can directly be sensed from the hardware interface or computed algorithmically by calculating the difference between the sensed encoderPosition in every dt interval.

The Kp, Ki and Kd parameters are updated as follows before the end of run deceleration zone:

(12) $$\begin{array}{cc} \; & \mathrm{If}\;\mathrm{velocity}\;\mathrm{is}\;\mathrm{too}\;\mathrm{low}\;(Vcurrent<Vmax-precision):\\ \; & \;\;\;\;\;\;\;\;\;\;\;\;Kp=Kp+dt/div\\ \; & \;\;\;\;\;\;\;\;\;\;\;\;Ki=Ki+dt/div \end{array}$$

(13) $$\begin{array}{cc} \; & \mathrm{If}\;\mathrm{velocity}\;\mathrm{is}\;\mathrm{too}\;\mathrm{high}\;(Vcurrent>Vmax+precision):\\ \; & \;\;\;\;\;\;\;\;\;\;\;\;\;Ki=Ki-dt/div \end{array}$$

(14) $$\begin{array}{cc} \; & \mathrm{If}\;\mathrm{velocity}\;\mathrm{is}\;\mathrm{correct}\;(Vcurrent==Vmax\pm precision):\\ \; & \;\;\mathrm{Do}\;\mathrm{nothing}\;->\;\mathrm{No}\;\mathrm{changes}\;\mathrm{applied}\;\mathrm{to}\;Kp,Ki\;\mathrm{and}\;Kd \end{array}$$

(15) $$\begin{array}{cc} \; & \mathrm{Throughout}\;\mathrm{the}\;\mathrm{entire}\;\mathrm{run},\;\mathrm{as}\;\mathrm{an}\;\mathrm{algorithmic}\;\mathrm{simplification}:\\ \; & \;\;\;\;\;\;\;\;\;\;\;\;Kd=Kp/3 \end{array}$$

For the end of run deceleration zone, the “holdCount” parameter also comes into the picture to augment the div value for Kp, Ki and Kd adjustments.

## 5. Results

To observe the operation of the proposed PID++ algorithm, many scenarios with different travel distances, speeds, trapezoidal acceleration/deceleration profiles, and weight quantities were tested on a single software compilation. Different load weights ranging from 0 g up to 1 Kg were used. For the purpose of illustration, the experimental results with 2 different weights are graphically demonstrated in this section of the paper. Further, a video demonstration (seen in the Supplementary Materials) has been produced, showing the running apparatus with 5 different weights.

### 5.1. Graphical Responses

The Distance Traveled (Encoder Position in cnts), Output (or Experiment Output) of the PID++ algorithm applied to the motor (represented as PWM values), and the Speed of the motion in each test scenario, are graphically shown in each horizontal 3-plot Figure of Section 5.1. On the axes, Time is shown in seconds, Position is shown in cnts and Speed is shown in cnts/ms.

All the plots corresponding to the velocity (Speed) graphs in Figure 13, Figure 14, Figure 15, Figure 16, Figure 17, Figure 18, Figure 19, Figure 20, Figure 21, Figure 22, Figure 23, Figure 24, Figure 25, Figure 26, Figure 27, Figure 28, Figure 29, Figure 30, Figure 31 and Figure 32 hereafter follow the four (4) main areas (as explained in a typical PID++ run depicted in Figure 4): Initial Acceleration Zone, followed by a Plateau Phase, followed by the End of Run Deceleration Zone, and then ending with a Search Phase.

**Figure 13:**
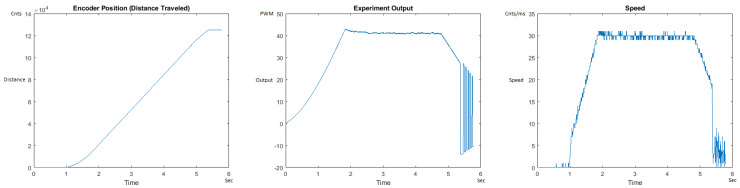
Encoder position, output and speed plots for PID++ UP operation—Vmax 30 cnts/ms, Adesired 0.055 cnts/ms${}^{2}$—10 g.

**Figure 14:**
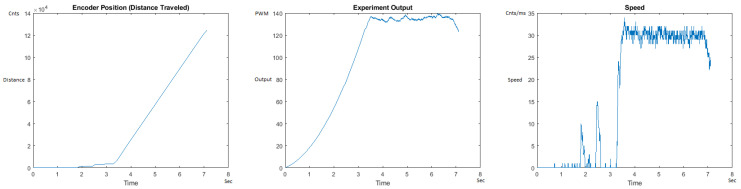
Encoder position, output and speed plots for PID++ UP operation—Vmax 30 cnts/ms, Adesired 0.055 cnts/ms${}^{2}$—1 Kg.

Figure 13 and Figure 14 begin the UP direction MATLAB output graphs of the PID++ as specified, with the plateau and trapezoidal values of of Vmax = 30 cnts/ms, Adesired = 0.055 cnts/ms${}^{2}$ using two different weights of 10 g and 1 Kg. Figure 13 Output graph shows the searching at the end of the run to find the correct Xdestination. Figure 14 Speed and Position graphs show the learning process involved in dealing with a relatively large weight to maintain specification.

**Figure 15:**
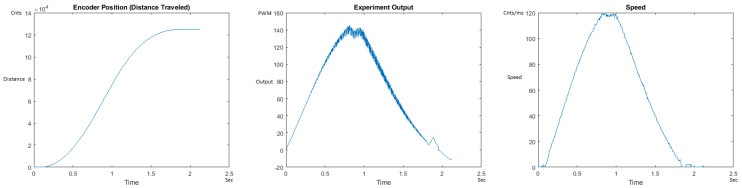
Encoder position, output and speed plots for PID++ UP operation—Vmax 120 cnts/ms, Adesired 0.040 cnts/ms${}^{2}$—10 g.

**Figure 16:**
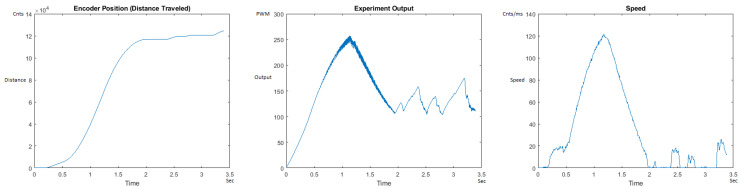
Encoder position, output and speed plots for PID++ UP operation—Vmax 120 cnts/ms, Adesired 0.040 cnts/ms${}^{2}$—1 Kg.

Figure 15 and Figure 16 show the MATLAB output of the PID++ as specified differently, with the correct plateau and trapezoidal values. Both Figure 15 and Figure 16 Output graphs show a small amount of searching at the end of the run to find the correct Xdestination. Figure 16 Speed and Position graphs show a decreased learning process involved with a larger weight due to the higher Vmax.

**Figure 17:**
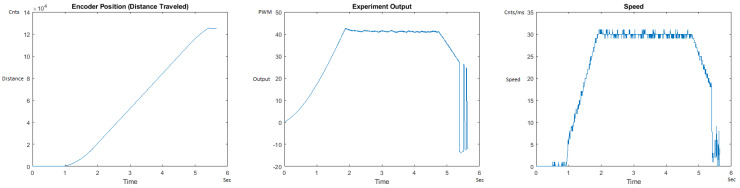
Encoder position, output and speed plots for PID++ UP operation—Vmax 30 cnts/ms, Adesired 0.015 cnts/ms${}^{2}$—10 g.

**Figure 18:**
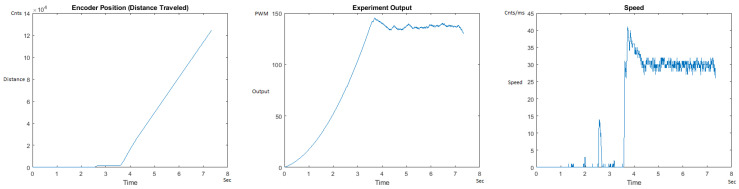
Encoder position, output and speed plots for PID++ UP operation—Vmax 30 cnts/ms, Adesired 0.015 cnts/ms${}^{2}$—1 Kg.

**Figure 19:**
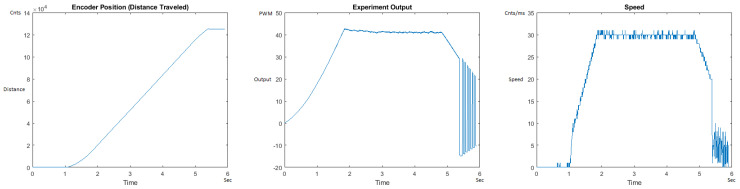
Encoder position, output and speed plots for PID++ UP operation—Vmax 30 cnts/ms, Adesired 0.040 cnts/ms${}^{2}$—10 g.

**Figure 20:**
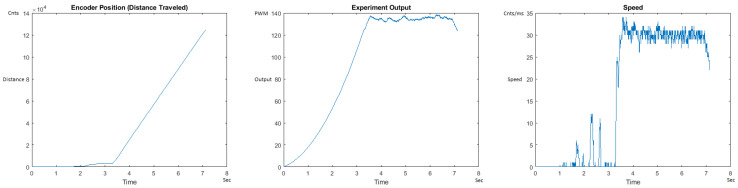
Encoder position, output and speed plots for PID++ UP operation—Vmax 30 cnts/ms, Adesired 0.040 cnts/ms${}^{2}$—1 Kg.

Figure 17 and Figure 18 show the MATLAB output of the PID++ as specified, with the new plateau and trapezoidal values of Vmax = 30 cnts/ms, Adesired = 0.015 cnts/ms${}^{2}$. Figure 19 and Figure 20 show the MATLAB output of the PID++ as specified, with other newly specified plateau and trapezoidal values. Figure 17 and Figure 19 Output graphs show the searching at the end of the run to find the correct Xdestination. Figure 20 Speed and Position graphs show the learning process involved in dealing with a relatively large weight to maintain specification.

**Figure 21:**
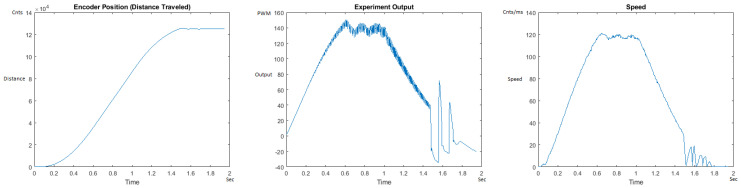
Encoder position, output and speed plots for PID++ UP operation—Vmax 120 cnts/ms, Adesired 0.055 cnts/ms${}^{2}$—10 g.

**Figure 22:**
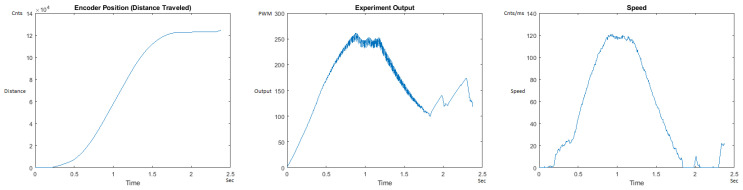
Encoder position, output and speed plots for PID++ UP operation—Vmax 120 cnts/ms, Adesired 0.055 cnts/ms${}^{2}$—1 Kg.

Figure 21 and Figure 22 show the MATLAB output of the remaining UP graphs of the PID++ as newly specified, with the correct plateau and trapezoidal values. Both Figure 21 and Figure 22 Output graphs show some searching at the end of the run to find the correct Xdestination. Figure 22 Speed and Position graphs show a decreased learning process involved with a larger weight due to the higher Vmax.

**Figure 23:**
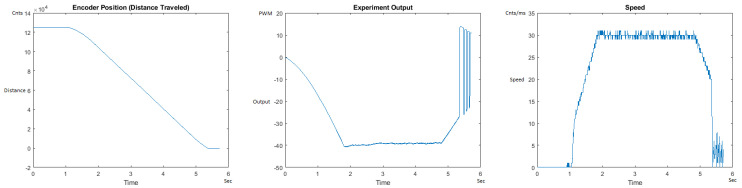
Encoder position, output and speed plots for PID++ DOWN operation—Vmax 30 cnts/ms, Adesired 0.015 cnts/ms${}^{2}$—10 g.

**Figure 24:**
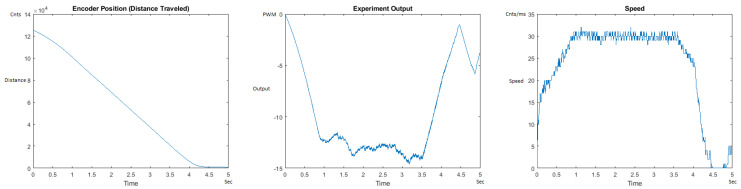
Encoder position, output and speed plots for PID++ DOWN operation—Vmax 30 cnts/ms, Adesired 0.015 cnts/ms${}^{2}$—1 Kg.

Figure 23 and Figure 24 begin the MATLAB output graphs in the DOWN direction. The decreased output level for the heavier weight can be noticed because of gravity in the DOWN direction. Figure 23 and Figure 24 show the MATLAB output of the PID++ as specified, with the correct plateau and trapezoidal values of Vmax = 30 cnts/ms, Adesired = 0.015 cnts/ms${}^{2}$. Figure 23 Output graph shows the searching at the end of the run to find the correct Xdestination. Figure 24 Speed and Position graphs show that the learning process is not involved with the DOWN direction because of gravity assistance.

**Figure 25:**
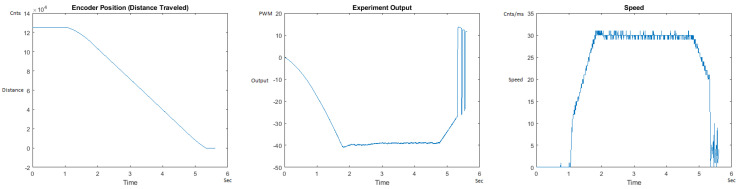
Encoder position, output and speed plots for PID++ DOWN operation—Vmax 30 cnts/ms, Adesired 0.040 cnts/ms${}^{2}$—10 g.

**Figure 26:**
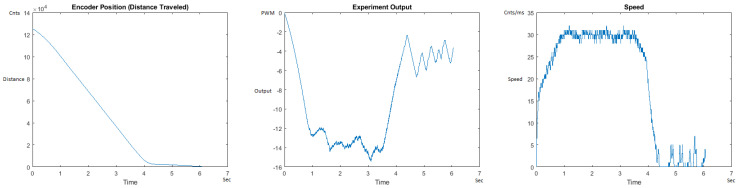
Encoder position, output and speed plots for PID++ DOWN operation—Vmax 30 cnts/ms, Adesired 0.040 cnts/ms${}^{2}$—1 Kg.

Figure 25 and Figure 26 show the MATLAB output of the PID++ as differently specified, with the correct plateau and trapezoidal values. Both Output graphs show the searching at the end of the run to find the correct Xdestination. Figure 26 Speed and Position graphs show that the learning process is not involved with the DOWN direction because of gravity assistance.

**Figure 27:**
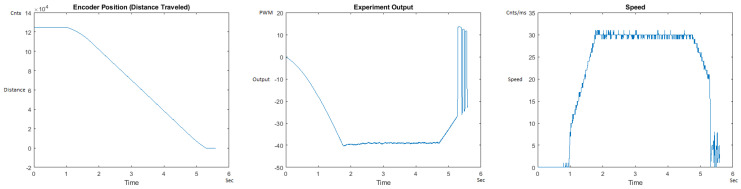
Encoder position, output and speed plots for PID++ DOWN operation—Vmax 30 cnts/ms, Adesired 0.055 cnts/ms${}^{2}$—10 g.

**Figure 28:**
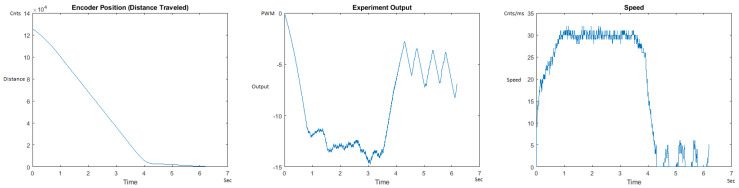
Encoder position, output and speed plots for PID++ DOWN operation—Vmax 30 cnts/ms, Adesired 0.055 cnts/ms${}^{2}$—1 Kg.

Figure 27 and Figure 28 show the MATLAB output of the PID++ as newly specified, with the correct plateau and trapezoidal values. Both Output graphs show the searching at the end of the run to find the correct Xdestination. Figure 28 Speed and Position graphs show that the learning process is not involved with the DOWN direction because of gravity assistance.

**Figure 29:**
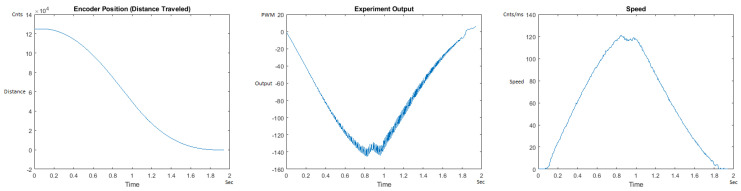
Encoder position, output and speed plots for PID++ DOWN operation—Vmax 120 cnts/ms, Adesired 0.040 cnts/ms${}^{2}$—10 g.

**Figure 30:**
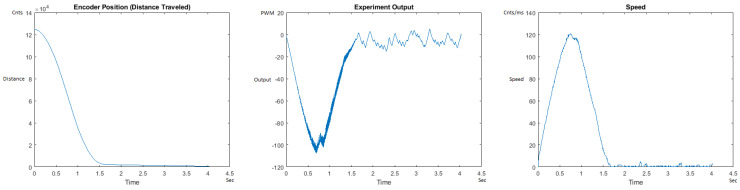
Encoder position, output and speed plots for PID++ DOWN operation—Vmax 120 cnts/ms, Adesired 0.040 cnts/ms${}^{2}$—1 Kg.

Figure 29 and Figure 30 show the MATLAB output of the PID++ differently specified, with the correct plateau and trapezoidal values. Figure 30 Output graph shows the searching at the end of the run to find the correct Xdestination. Figure 30 Speed and Position graphs show that the learning process is not involved with the DOWN direction because of gravity assistance.

**Figure 31:**
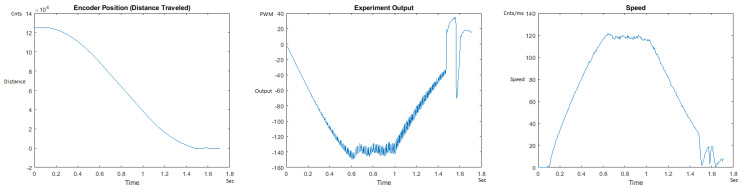
Encoder position, output and speed plots for PID++ DOWN operation—Vmax 120 cnts/ms, Adesired 0.055 cnts/ms${}^{2}$—10 g.

**Figure 32:**
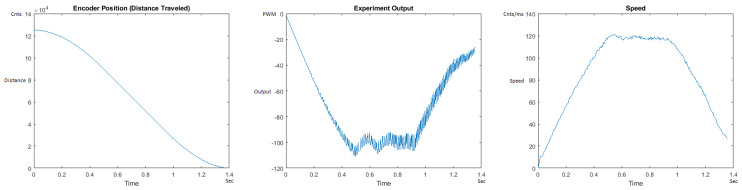
Encoder position, output and speed plots for PID++ DOWN operation—Vmax 120 cnts/ms, Adesired 0.055 cnts/ms${}^{2}$—1 Kg.

Figure 31 and Figure 32 show the MATLAB output of the PID++ as newly specified, with the correct plateau and trapezoidal values. Figure 31 Output graph shows the searching at the end of the run to find the correct Xdestination. Figure 32 Speed and Position graphs show that the learning process is not involved with the DOWN direction because of gravity assistance.

**Figure 33:**
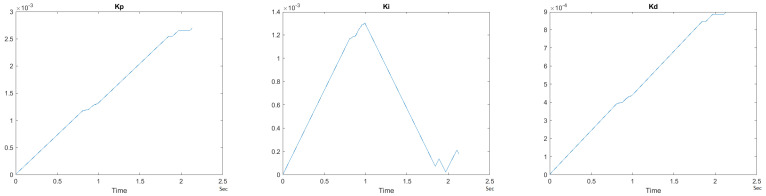
Kp, Ki and Kd parameters for PID++ UP–Vmax 120 cnts/ms, Adesired 0.055 cnts/ms${}^{2}$—10 g.

**Figure 34:**
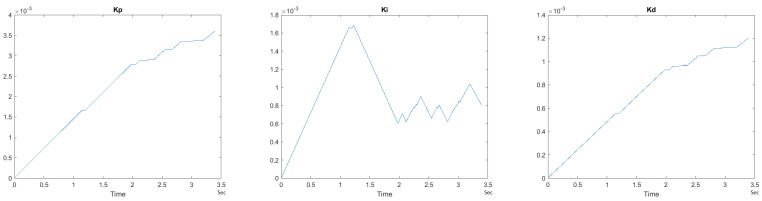
Kp, Ki and Kd parameters for PID++ UP–Vmax 120 cnts/ms, Adesired 0.055 cnts/ms${}^{2}$—1 Kg.

To observe the dynamic nature of the PID++ coefficients, Kp, Ki and Kd values are plotted with respect to time for a sample scenario run of Vmax = 120 cnts/ms, Adesired = 0.055 cnts/ms${}^{2}$ for the two weight loads of 10 g and 1 Kg in the UP direction. Figure 33 and Figure 34 show the graphs of these parameters.

It is clearly seen that the Kp, Ki and Kd parameters of PID++ do not remain constant and periodically change to adjust the motion to the desired trapezoidal profile operation.

Overall, from the graphical responses, it is clearly observed that in all runs with PID++, the desired distance traveled (0–125,000 cnts) in the UP direction and (125,000–0 cnts) in the DOWN direction, and the user specified Vmax with the desired acceleration/deceleration were correctly achieved from the plateau and trapezoidal profiles for different weight loads.

In addition, the Output graphs clearly show larger values required for the heavier weight in the UP direction. In the DOWN direction, as gravity also comes into picture to assist the motion, the Output graphs show negative responses where higher values are again correctly observed for the larger weight.

### 5.2. PID++ Comparison to Other Approaches

To compare the PID++ operation with other lightweight motion control mechanisms, the basic PID was selected.

**Figure 35:**
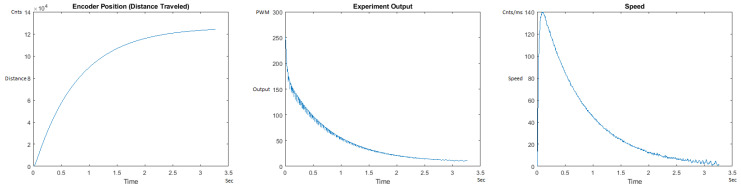
Encoder position, output and speed plots for basic PID UP operation—10 g.

**Figure 36:**
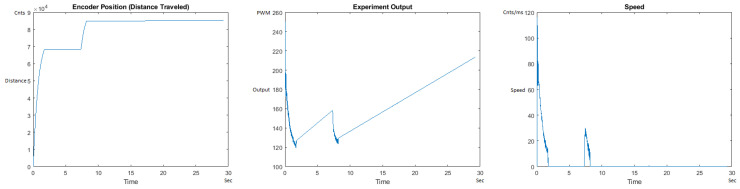
Encoder position, output and speed plots for basic PID UP Operation—1 Kg.

**Figure 37:**
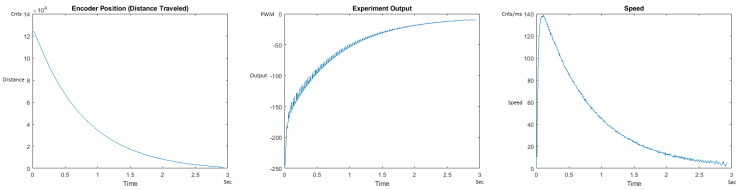
Encoder position, output and speed plots for basic PID DOWN operation—10 g.

**Figure 38:**
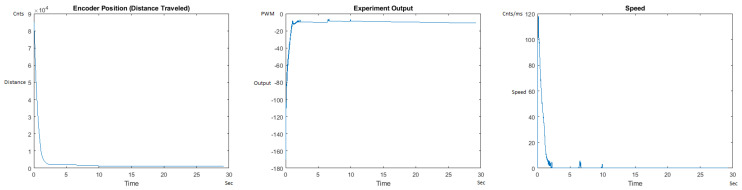
Encoder position, output and speed plots for basic PID DOWN operation—1 Kg.

Figure 35 and Figure 36 show the MATLAB output for the basic/standard PID in the UP direction with 10 g and 1 Kg and its poor response. As can be seen, the destination of 125,000 cnts is not reached and/or the speed and acceleration are lacking control, showing that the basic PID needs readjustment of coefficients for different weights. Figure 37 and Figure 38 also show the basic PID with 10 g and 1 Kg in the DOWN direction and its poor response. No symmetrical/trapezoidal profile is observed in the runs. The basic PID clearly fails to provide a controlled motion or even reach the specified destination when changing the load/weights.

Unlike every other approach for intelligent motion control including (1) Basic PID, (2) Single Neuron (Deep Learning), (3) Fuzzy Logic, (4) Classic Self-tuning and (5) Model Reference (Laplace Transform), the PID++ algorithm requires no pre-knowledge of the system of any kind, just like humans do all the time. Furthermore, no computationally costly math is required and therefore the system is able to run with ease on a standard microcontroller and not a DSP which most of the other techniques (2–5) require. The PID++ algorithm (unlike all of the other techniques) also comes with complete run motion control included, and therefore, can be implemented with a single command line entry:

e.g., (**call PID++(125,000, 120.0, 0.045, 0.005, 0.01**);

This command is all that is required to run the motor from the current location to encoder position 125,000 at a speed of 120.0 counts/ms, a symmetrical trapezoidal acceleration/deceleration profile of 0.045 counts/ms${}^{2}$, a dtmin of 5 ms, and a motion precision of 1%.

### 5.3. Computational Complexity Analysis

The computational complexity of the PID++ algorithm in Big O notations is O(n/(dtmin×1000)), where *n* refers to the total number of encoder position samples (which is taken every 1ms) and dtmin is some multiple of this sample rate. This is while neural networks require $O(n^{4})$ for forward propagation and $O(n^{5})$ for back propagation (*n* being a parameter of the network structure, e.g., number of layers/neurons, or iterations).

As no other algorithm including (1) Basic PID, (2) Single Neuron (Deep Learning), (3) Fuzzy Logic, (4) Classic Self-tuning and (5) Model Reference (Laplace Transform) is even capable of complete run motion control, as is the case with the PID++, no comparable Big O value for these different other approaches can be put forth for a comparison.

### 5.4. Limitations

When the PID++ algorithm is applied and used properly, the results are quite remarkable, as shown and described in this paper. To obtain this though, certain simple electrical and mechanical guidelines must be followed:

1. The motor must be properly sized for the amount of load required to be moved or lifted.
2. Adesired values must be set appropriately and reasonably for the Vmax speed specified for the run, otherwise Vmax may never be reached.
3. Vmax should be set to reasonable values depending on the size of the motor and the given mass to be moved or lifted in the run.
4. Xdestination should be a reachable value.
5. The motor power supply should be sized properly for the motor size and size of the masses to be moved or lifted.
6. For large loads lifted by relatively small motors, the ENCODER_TARGET_COUNT will need to be enlarged to allow the motor to more easily find the Xdestination. This is just like human behavior.

### 5.5. PID++ Algorithm Applications

The PID++ algorithm is applicable to a variety of sectors:

1. Commercial: printers, toys, appliances, etc.
2. Industrial: Computer Numeric Control (CNC) machines, 3D printers, robotics, general motion control.
3. Biomedical: bionics, prosthetics, artificial limbs, artificial implants, robotic surgery.
4. Space: robotic landers, Lunar and Martian Geological Exploration.

All of these applications, no matter what sector it pertains to, will require accurate, adaptive, low computational cost, linear motion control. The PID++ algorithm is useful in all of these cases, with or without load variations.

## 6. Conclusions and Future Directions

The PID++ algorithm uses minor adjustments in tuning on a periodic basis to achieve extremely precise, humanoid motion control. Whereas most PID controllers require that the transfer function of the process be known and that the PID coefficients be configured for that specific process, generally represented as the Laplace transform, the proposed PID++ algorithm can operate with any linear motion control process, without any foreknowledge of the system transfer function and regardless of load.

The proposed algorithm shows that human-like motion control is possible and with accuracy and precision only obtainable with a computer-controlled system. Additionally, it is demonstrated to be computationally lightweight as it successfully executes with precision and speed on just an Arduino Uno.

Many different travel distances, speeds, trapezoidal acceleration/deceleration profiles, and weight quantities were tested with a single software executable, and demonstrated to operate successfully.

In the future, testing with larger electric motors and heavier loads will be performed. Moreover, the PID++ algorithm will be tested with other types of propulsion systems with a Pulse Width Modulation (PWM) input as well. Furthermore, beta testing with commercial, industrial, biomedical, and space applications will be planned out and pursued.

## Data Availability

The data supporting the reported results presented in this study have been created by the authors and are openly available in the Code Ocean public repository, at https://doi.org/10.24433/CO.6585291.v2.

## Supplementary Materials

The following are available online at https://www.mdpi.com/1424-8220/21/2/456/s1. A video demonstration of the PID++ algorithm has been produced, showing the running apparatus with 5 different weights.
