# Supplementary material for: PID++: A Computationally Lightweight Humanoid Motion Control Algorithm
Source: Sensors (Basel). 2021 Jan 11;21(2):456. doi: 10.3390/s21020456 (PMC7826970; doi:10.3390/s21020456)
Supplement: Supplementary file 1 [file sensors-21-00456-s001.pdf]

sensors

MDPI

Supplementary document of Article

PID++: A Computationally Lightweight Humanoid Motion Control Algorithm

Thomas F. Arciuolo [1] and Miad Faezipour[1,2] *

[1] Department of Computer Science and Engineering, University of Bridgeport, 221 University Avenue, Bridgeport, CT 06604, USA; tarciuol@my.bridgeport.edu
[2] Department of Biomedical Engineering, University of Bridgeport, 221 University Avenue, Bridgeport, CT 06604, USA
* Correspondence: Correspondence: mfaezipo@bridgeport.edu; Tel.: +1-203-576-4702

A video demonstration of the PID++ algorithm has been produced, showing the running apparatus with 5 different weights:
https://drive.google.com/file/d/1TxrR1KVJrK9-MQwQ7g2V7SHqIMLap8kk/view?usp=sharing
